# Dynamics of Anti-SARS-CoV-2 IgA and IgG Responses and Their Protective Effect against Fatal Disease after Booster COVID-19 Vaccination

**DOI:** 10.3390/vaccines12010012

**Published:** 2023-12-21

**Authors:** Matthaios Speletas, Ioanna Voulgaridi, Zacharoula Bogogiannidou, Styliani Sarrou, Maria A. Kyritsi, Aikaterini Theodoridou, Katerina Dadouli, Alexia Matziri, Alexandros Vontas, Dimitra Pappa, Adamos-Konstantinos Konstantinou, Christina Tsigalou, Fani Kalala, Varvara A. Mouchtouri, Christos Hadjichristodoulou

**Affiliations:** 1 Department of Immunology & Histocompatibility, Faculty of Medicine, University of Thessaly, 41500 Larissa, Greece; maspel@med.uth.gr (M.S.); ssarrou@uth.gr (S.S.); katetheod41222@gmail.com (A.T.); fkalala@uth.gr (F.K.); 2 Laboratory of Hygiene and Epidemiology, Faculty of Medicine, University of Thessaly, 41222 Larissa, Greece; ioavoulg@uth.gr (I.V.); zbogogiannidou@uth.gr (Z.B.); mkiritsi@uth.gr (M.A.K.); adadouli@uth.gr (K.D.); alexmatz@uth.gr (A.M.); avontas@uth.gr (A.V.); mouchtourib@uth.gr (V.A.M.); 3 Department of Pathology, Faculty of Medicine, University Hospital of Larissa, 41500 Larissa, Greece; dimipap2@gmail.com; 4 Psychogeriatric Hospital “Ippokrateio Therapeutirio”, 40011 Larissa, Greece; konstadam@gmail.com; 5 Laboratory of Hygiene and Environmental Protection, Democritus University of Thrace, Dragana Campus, 68100 Alexandroupolis, Greece; ctsigalo@med.duth.gr

**Keywords:** booster vaccination, COVID-19 mRNA vaccination, anti-SARS-CoV-2 IgA responses, anti-SARS-CoV-2 IgG responses, SARS-CoV-2

## Abstract

During the post-coronavirus disease (COVID-19) era, a primary question is whether booster vaccination is effective against severe COVID-19 and should be recommended, particularly to individuals at high risk for severe disease (i.e., the elderly or those with additional severe comorbidities). From December 2020 to February 2023, a cohort study was conducted to estimate IgG and IgA immunogenicity and the dynamics of booster mono- and bivalent COVID-19 mRNA vaccines in 260 individuals (male/female: 114/146, median age: 68 years, interquartile range (IQR) = 31) who initially received either mRNA (218) or adenovirus-vector-based vaccines (42). Participants were followed until the 90th day after the third booster dose. Our cohort study indicated a beneficial effect of booster vaccination on the magnitude of IgG and IgA severe acute respiratory syndrome coronavirus 2 (SARS-CoV-2) antibodies. We found that second and third booster doses were more protective than one against fatal disease (*p* = 0.031, OR 0.08). In conclusion, booster COVID-19 vaccination should be strongly recommended, especially to individuals at high risk for severe/fatal disease.

## 1. Introduction

From early 2020, the coronavirus disease (COVID-19) pandemic dramatically affected human health and economic activities globally. It should be noted that timely and widespread COVID-19 vaccination strategies were implemented. Although the initial COVID-19 vaccination was not proven to be sterilizing, it was shown to be protective against severe diseases, markedly reducing the incidence of hospitalization and fatality [1,2,3,4,5,6]. The effectiveness of the COVID-19 vaccines was affected by the high mutagenicity of severe acute respiratory syndrome coronavirus 2 (SARS-CoV-2) and the emergence of new viral variants, which could escape vaccine-derived immunity [3]. Therefore, there was a need to develop updated vaccines that could offer higher and more durable protection against new circulating variants [7]. Thus, a primary question was whether vaccination with the updated vaccines remained effective against the rising SARS-CoV-2 variants and should be recommended, particularly to individuals at high risk for severe disease, such as the elderly or patients with additional severe comorbidities. In this context, recent studies have demonstrated a significant benefit from administration of booster COVID-19 vaccination, related to protection against current SARS-CoV-2 variants [3,4,5,6].

In our observational study, we examined the immunogenicity and effectiveness of booster vaccination with monovalent mRNA and bivalent vaccines against COVID-19. The purpose of the current study was to investigate the immunogenicity of booster vaccination induced by an initial monovalent mRNA vaccine followed by additional monovalent or bivalent boosters against COVID-19 by examining the induced anti-SARS-CoV-2 IgG and IgA antibodies; and to examine the effectiveness of repeated booster vaccination in reducing the risk of severe disease and/or death from disease.

## 2. Materials and Methods

### 2.1. Study Design and Population

From January 2021 to February 2023, a prospective cohort study was conducted involving repeated blood collection at specific timepoints after each vaccination (detailed below) from 260 Greek individuals whose initial vaccination regimen included mRNA (BNT162b2 vaccine: Comirnaty^®^; BioNTech/Pfizer, Mainz, Germany) or adenovirus-vector-based vaccines (ChAdOx1 nCoV-19:AZD1222 of Oxford/AstraZeneca, University of Oxford, UK, and Ad26.COV2.S: Janssen Biontech, Inc., Janssen Pharmaceutical company, Johnson & Johnson, New Brunswick, NJ, USA). The majority of enrolled individuals were elderly residents of long-term care facilities who displayed severe comorbidities. The rest of the participants in the sample were derived from the general population and healthcare workers (HCWs) who were randomly selected. This sample was derived from a larger study population and was collected with the sole criterion of a history of at least one booster dose. It should be mentioned that a booster dose was defined as any dose following the initial completed immunization, considering first vaccine type. Administration of vaccination doses was in line with national vaccination strategies, considering circulating SARS-CoV-2 variants. 

We considered participants with a positive polymerase chain reaction (PCR) result from a nasopharyngeal swab and/or positive anti-N IgG antibody titers as having a positive history of SARS-CoV-2 infection. We recorded data related to the study population characteristics, history and number of SARS-CoV-2 infections, severity of infections and administration of antiviral treatment, vaccination coverage against COVID-19, and chronic sequence of every participant’s SARS-CoV-2 infection and vaccination. For the analysis, the severity of SARS-CoV-2 infection was categorized as “1” in case of asymptomatic or mild symptoms, and “2” in case of hospitalization and death. Deaths attributed to another cause unrelated to COVID-19 were recorded in terms of follow up, but were not included in COVID-19 deaths. 

Serum sampling was performed in all participants at day 21, 42, 90, and 180 following the initial vaccination; at day 90 and 180 after the first and second booster doses; and at day 21 and 90 after the third booster dose. The duration of the follow-up period was defined as the day of the first recorded dose of initial vaccination until day 90 following the last recorded third booster dose. Only data on day 90 and 180 following the first and second booster doses and on day 21 and 90 following the third booster dose were analyzed for IgG antibodies; outcomes derived from analysis of previous sampling days have already been published [8,9]. Regarding IgA antibodies, only data on day 90 were included because on this day individuals already display low IgA antibody levels, and their titers are expected to further decrease after day 90 [8]. Anti-S and anti-N anti-SARS-CoV-2 IgG and IgA antibodies were quantified by chemiluminescent microparticle immunoassay (CMIA), with an ARCHITECT i2000SR immunoassay analyzer (Abbott, Abbott Park, IL, USA) using SARS-CoV-2 IgG II Quant Reagent Kit and SARS-CoV-2 IgG Reagent Kit, and enzyme-linked immunosorbent assay (ELISA) using a SERION ELISA agile SARS-CoV-2 IgA ESR400A kit (Serion, Würzburg, Germany) according to the manufacturer instructions, as described in [8]. 

### 2.2. Statistical Analysis 

Statistical analysis was performed with SPSS Statistics for Windows, Version 29.0 (IBM Corp., Armonk, NY, USA) and GraphPad Prism (Version number 10.1.1) software. Analysis of continuous variables was conducted using the Mann–Whitney U test and Spearman’s correlation coefficient, while data were checked for deviation from normal distribution using the Shapiro–Wilk normality test. Kaplan–Meier curves were used to estimate the probability of infection at different timepoints, and a log-rank test was employed to assess differences between covariates. Multiple regression was used to determine independent predictors of antibody quantities/levels. Binary logistic regression and cox regression were used to determine independent predictors of infection, severity, and death. Variables with *p*-value < 0.2 on univariate analysis were included in multivariate analysis. A 5% significance level was set for all analyses.

### 2.3. Ethical Statement

All participants or relatives of individuals with mental disorders provided informed consent. The study was conducted according to the principles of the Helsinki Declaration and was approved by the ethical committee of the University of Thessaly (No. 4/29.01.2021).

## 3. Results

Of the 260 participants in our study, 146 were women (female/male: 146/114) with a median age of 68 years (range: 26–107 years, interquartile range (IQR) = 31 years). A total of 167 (64.2%) individuals were residents in long-term care facilities, while 47 (18.1%) were HCWs and 46 (17.7%) came from the general population. The demographics and comorbidities of our study population are detailed in Supplementary Table S1.

From our study population, 218 participants were initially vaccinated with BNT162b2 (Comirnaty; Pfizer/BioNtech) (female/male: 129/89, median age: 72 years, range: 27–107 years, IQR = 30.5), and 42 with adenovirus-vector-based vaccines (female/male: 17/25, median age: 50 years, range: 26–84 years, IQR = 17.75). Specifically, 24 received ChAdOx1 nCoV-19 from Oxford/AstraZeneca and 18 received Ad26.COV2.S from Johnson & Johnson’s Janssen. All participants received a first booster vaccination with BNT162b2; 23 died from medical/pathological causes unrelated to COVID-19, while four died due to COVID-19. Out of the remaining sample (N = 233), 141 were vaccinated with a second booster dose (60.5%, female/male: 75/66, median age: 74 years old). Of these, 100 received BNT162b2 (70.9%, female/male: 51/49, median age: 75 years old); 15 received the bivalent BA.1 vaccine (Comirnaty Original/Omicron BA.1) (10.6%, female/male: 6/9, median age: 73 years old); and 26 received bivalent BA.4/5 (Comirnaty Original/Omicron BA.4–5) (18.5%, female/male: 18/8, median age: 61.5 years old). Out of 233 participants, 8 died due to medical/pathological causes unrelated to COVID-19, while 1 participant died due to COVID-19. All nine participants who died were vaccinated with a second booster dose. Of the remaining 224 participants who had received two booster doses, a total of 52 had also received a third booster dose (23.2%, female/male: 30/22, median age: 78 years old). Supplementary Table S2 details the characteristics of the population who died due to COVID-19.

**Figure 1:**
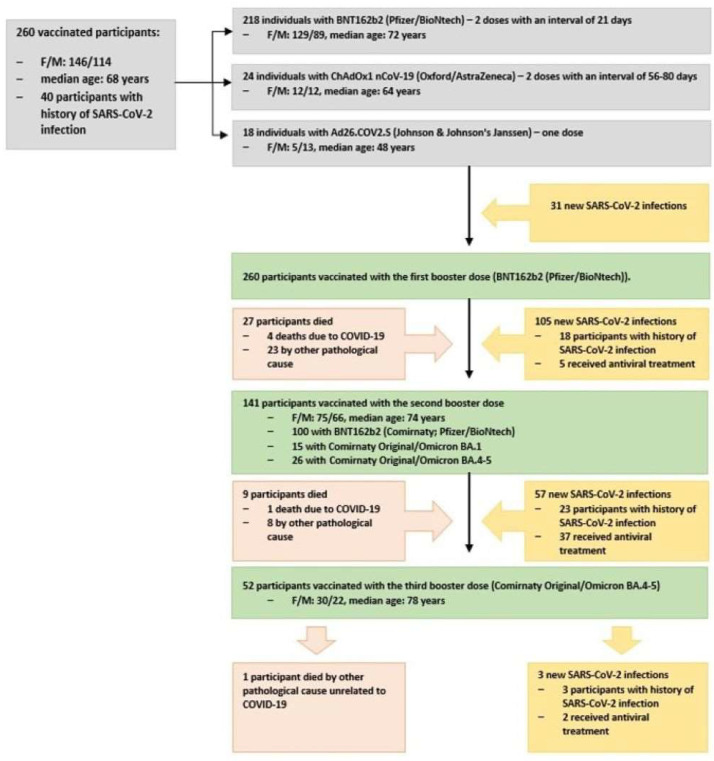
Participants’ characteristics, course of their COVID-19 vaccination status, and medical history of SARS-CoV-2 infection and treatment.

Forty participants (15.4%) had been infected by SARS-CoV-2 at least 3 months prior to their initial vaccination. An additional 31 SARS-CoV-2 infections occurred prior to the first booster dose. Among those vaccinated with the first booster (Ν = 260), 105 participants were diagnosed with SARS-CoV-2 infection; 5 of them required oral antiviral treatment (nirmatrelvir/ritonavir) and 18 had a positive medical history for SARS-CoV-2 infection. Among those vaccinated with a second booster (N = 141), 57 were diagnosed with SARS-CoV-2 infection, 37 of whom received antiviral drugs orally and 23 had a positive medical history of SARS-CoV-2 infection. Among those vaccinated with a third booster (N = 52), 3 were infected by SARS-CoV-2 and had a positive medical history of SARS-CoV-2 infection. Two of these participants were administered tablets of nirmatrelvir/ritonavir. The characteristics of our study population, the course of COVID-19 vaccination status, and the medical history of SARS-CoV-2 infection and treatment are depicted in Figure 1. 

Among the fully vaccinated population (only by initial immunization) infected by SARS-CoV-2 (N = 32) prior to administration of booster doses, no one developed severe disease. Thus, nobody was categorized as severity 2. Regarding confirmed infections among those who received one booster dose (N = 105), 9 presented with critical disease and 4 died. Among the infected participants with a history of two booster doses (N = 57), 3 were characterized with severity 2, and 1 died. No participants who received a triple booster dose and were infected by SARS-CoV-2 presented with severity 2.

**Figure 2:**
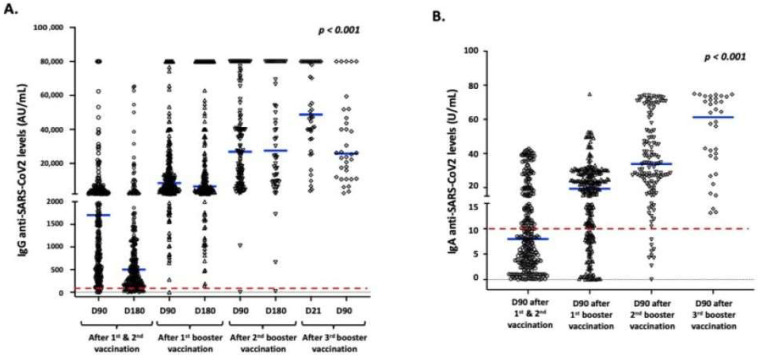
Magnitude and dynamics of IgG (**A**) and IgA (**B**) responses specific to SARS-CoV-2 in study participants. Blue lines indicate median values, and red dotted lines represent the cutoff of positive anti-SARS-CoV-2 IgG (50 AU/mL) and IgA (10 U/mL). In the X-axis the ”D” refers to day and the following number defines the number of days after vaccination.

Figure 2 depicts the magnitude and dynamics of IgG and IgA anti-SARS-CoV-2 antibodies at specific timepoints. The results are derived from all participants vaccinated with each booster dose. Booster vaccination resulted in a statistically significant increase in anti-SARS-CoV-2 IgG and IgA responses (*p*-value < 0.001); this finding was more prevalent for IgA levels, which increased continuously after each booster dose. Particularly for IgG levels, a significant increase was detected on day 90 and 180 following the first booster compared with initial vaccination. A further increase was observed after the second booster at both timepoints. The high level of IgG responses was preserved on day 90 following the third booster vaccination. 

We conducted both univariate and multivariate analyses in order to examine the correlation between the magnitude of anti-SARS-CoV-2 IgG, comorbidities, history of SARS-CoV-2 infection, and type of previously administrated vaccine. These analyses were performed for the magnitude of IgG and IgA antibody titer on the 90th day after the first, second, and third booster dose. The results for the IgG antibodies on the 90th day following the first booster dose are provided in Supplementary Table S3, the results for the IgG antibodies on the 90th day after the second booster dose are provided in Supplementary Table S4, and the results for the IgG antibodies on the 90th day after the third booster dose are provided in Supplementary Table S5. Supplementary Tables S6–S8 provide the results for IgA at the corresponding timepoints (Tables S3–S8). The magnitude of IgG and IgA responses on the 90th day following the first and second booster was significantly associated with a history of SARS-CoV-2 infection, the type of initial vaccine, and the type of second booster. The presence of comorbidities was observed to statistically significantly affect the magnitude of anti-SARS-CoV-2 IgA by reducing its levels only on day 90 following the first booster dose. These outcomes are shown in Supplementary Materials (Table S9). 

Following the analysis, we included comorbidities estimated as factors with a *p*-value < 0.2 in a univariate analysis. We repeated the multivariate analysis testing the correlation between the magnitude of anti-SARS-CoV-2 IgG, comorbidities, type of initial vaccination, and type of second booster dose in two groups: (i) participants with a positive history of one SARS-CoV-2 infection; and (ii) participants with a negative history of SARS-CoV-2 infection. In this analysis, a sample with a history of two SARS-CoV-2 infections was excluded due to too few participants. The age, sex, and selected comorbidities were not statistically significant factors affecting the magnitude of IgG antibodies on day 90 following the first and second booster dose in either study group (Table 1). Following the first booster dose on day 90, participants who had initially received an mRNA vaccine developed a higher titer of antibodies than those who had been immunized with adenovirus-vector-based vaccines. This difference was statistically significant in the group with a positive history of one SARS-CoV-2 infection (group with positive history: immunized with adenovirus-vector-based vaccines vs. with mRNA: median of 9022 AU/mL with an IQR of 11,563 AU/mL vs. median of 23,941 AU/mL with IQR of 36,924 AU/mL, which are significantly different (*p*-value = 0.015); group with negative history: immunized with adenovirus-vector-based vaccines vs. with mRNA: median of 3637 AU/mL with IQR of 5396 AU/mL vs. median of 6456 AU/mL with IQR of 9873 AU/mL, with no significant difference (*p*-value = 0.130)) (Table 1A). On day 90 following the second booster, the bivalent vaccines induced a higher magnitude of IgG antibodies in both groups than the monovalent vaccine. The calculated difference between the titers was statistically significant in the group with a negative history of SARS-CoV-2 infection (group with positive history: vaccinated with bivalent vaccine vs. monovalent: median of 49,241 AU/mL with IQR of 51,723 AU/mL vs. median of 40,000 AU/mL with IQR of 61,535 AU/mL, with no significant difference (*p*-value = 0.488); group with negative history: vaccinated with bivalent vaccine vs. monovalent: median of 40,000 AU/mL with IQR of 43,746 AU/mL vs. median of 6519 AU/mL with IQR of 9949, which are significantly different (*p*-value = 0.001)) (Table 1Β).

We conducted a multivariable analysis by using the same criteria and aiming to examine the correlation of IgA concentration with comorbidities, type of initial vaccination, and type of second booster dose in the same two groups. The magnitude of IgA was not statistically significantly affected by age, sex, or underlying diseases, excluding participants with a medical history of stroke and transient ischemic attacks, who exhibited a lower IgA concentration (*p*-value = 0.048) (Table 2). Regarding the magnitude of IgA on day 90 following the first booster dose, it was statistically significantly higher in those initially vaccinated with mRNA and without a history of SARS-CoV-2 infection (group with negative history: immunized with adenovirus-vector-based vaccines vs. with mRNA: median of 5.6 U/mL with IQR of 10.2 U/mL vs. median of 17.1 U/mL with IQR of 19.3 U/mL, which are significantly different (*p*-value < 0.001)) (Table 2A). A statistically significant difference in the titer of IgA, in comparing the type of initial vaccination, was not found (Table 2A). On day 90 following the second booster dose, those with a history of one SARS-CoV-2 infection exhibited a higher titer of IgA with a statistically significant difference (group with positive history: vaccinated with bivalent vaccine vs. monovalent: median of 27.2 U/mL with IQR of 6.32 U/mL vs. median of 53.2 U/mL with IQR of 36.6 U/mL, which are significantly different (*p*-value < 0.001)) (Table 2B). The type of second booster dose did not cause a significant difference in the group with a negative history of SARS-CoV-2 infection (group with negative history: vaccinated with bivalent vaccine vs. monovalent: median 27.8 U/mL with IQR of 40.8 U/mL vs. median of 15.6 U/mL with IQR of 22.5 U/mL, with no significant difference (*p*-value = 0.347)) (Table 2B).

We further performed a Cox regression analysis on the participants who received first or second/third booster doses to investigate the impact of the number of booster doses on the participants’ survival (Table 3). A protective role of the second/third booster and a history of SARS-CoV-2 infection were observed without a statistically significant difference (Hazard Ratio (HR) = 0.77, *p*-value = 0.712 and HR = 0.55, *p*-value = 0.449, respectively). The presence of comorbidities and aging were found to be correlated with severity 2, but no statistically significant difference was found (HR = 2.63, *p*-value = 0.172 and HR = 1.03, *p*-value = 0.088, respectively) (Table 3).

**Figure 3:**
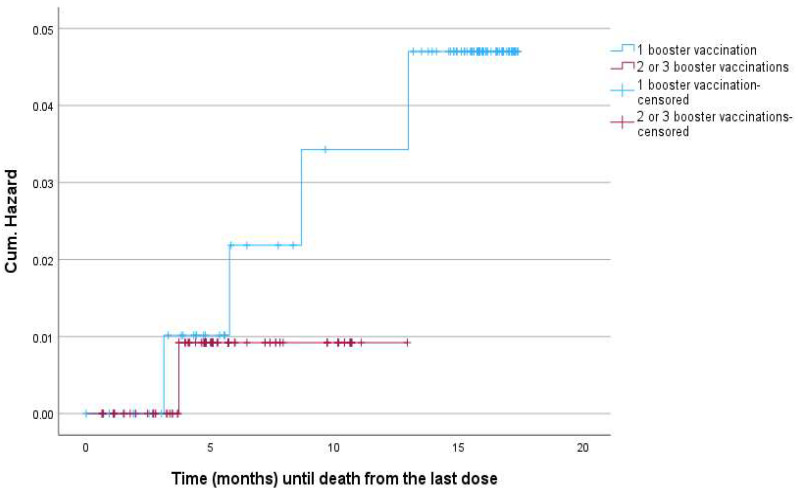
Reverse Kaplan–Meier survival curve considering the number of booster vaccination doses.

Using logistic regression, we investigated the effect of age, the presence of more than two comorbidities, and the number of booster doses on the risk of death due to COVID-19. Aging and comorbidities increased the probability of a fatal outcome, with no statistically significant differences (Odds Ratio (OR) = 1.05, *p*-value = 0.083 and OR = 2.77, *p*-value = 0.318, respectively). Two/three booster doses statistically significantly reduced the risk of COVID-19 death in comparison with only one booster (OR = 0.08, *p*-value = 0.031) (Table 4). This protective role was maintained even when we added the administration of antiviral treatment as an independent factor for the outcome (OR = 0.04, *p*-value = 0.046). According to the log-rank test (*p* = 0.513), no statistically significant correlation was found between the time of the administration of the last booster dose and the event of death (Figure 3) [10]. 

## 4. Discussion

Our cohort study indicated a beneficial effect of booster vaccination on the magnitude of anti-SARS-CoV-2 IgG and IgA antibodies. Similar outcomes have been found by other studies [11,12,13,14,15,16,17]. Each additional dose contributed cumulatively and maintained a high concentration of existing antibodies [15,16,17,18]. Our findings show that this beneficial effect was greater on IgA responses. To our knowledge, there are few studies that estimate the magnitude and dynamics of IgA, especially after three booster doses [14,18]. 

The phenomenon of a positive impact of booster doses on induced antibody responses was reinforced in cases of a positive history of SARS-CoV-2 infection and/or initial vaccination with an mRNA vaccine. In the literature, it can be seen that, via hybrid immunity, a higher magnitude of antibodies is recorded after booster vaccination doses [19]. Additionally, homologous vaccination with three doses of mRNA induces higher antibody titers than heterologous vaccination (initially two doses of adenovirus-vector-based vaccine followed by mRNA) [20]. 

A second booster vaccination with bivalent mRNA vaccines resulted in a higher IgG anti-SARS-CoV-2 antibody titer than with a monovalent vaccine, especially in those with a negative SARS-CoV-2 infection history. In contrast to the anti-SARS-CoV-2 IgG antibody titer, second booster doses with a monovalent vaccine induced a higher magnitude of IgA than with a bivalent vaccine. Examining the recent literature, no statistically significant difference in immunogenicity was noted between monovalent and bivalent vaccines as booster doses; these outcomes were based on neutralization assays [21,22]. Considering the above, booster vaccination against COVID-19 should be recommended with any available vaccine.

Vaccinated participants with two or three booster doses were at lower risk of death than those who received one booster dose. Previous studies have shown that booster vaccination resulted in increased IgG responses against SARS-CoV-2, reducing the risk of severe disease and death by breakthrough SARS-CoV-2 infections [23,24]. A second booster dose was associated with a 21% risk difference for COVID-19-related death compared with one booster dose [25]. Recent studies indicate that booster mRNA vaccination against COVID-19 results in a class switch towards non-inflammatory IgG4 antibodies, with a reduced capacity to mediate antibody-dependent phagocytosis and complement deposition [26,27]. Considering the aforementioned studies and our findings, we speculate that the protective effect of mRNA booster vaccination may be attributed to robust anti-spike IgA responses that increased over time, exerting a protective effect against fatal COVID-19. Our hypothesis is supported by another published study in which it was claimed that IgA antibodies play a significant role in immunity against COVID-19 [28]. Further relevant studies should be conducted to clarify this important issue.

A decrease in IgG antibody concentration was observed on day 90 compared with day 21 after the third booster dose. The final titer was similar to the induced titer at day 90 and 180 after the second booster. As only few published studies have investigated the course of antibodies after five vaccine doses, there is no clear explanation for this finding. A further longitudinal analysis of antibody response in larger cohorts is needed [16].

Our study presents some limitations. We did not conduct neutralization assays, given the questionable role of neutralizing antibodies in COVID-19 outcome [29]; thus, we could not estimate the proportion of neutralizing antibody titers of total virus-specific antibodies from the detected titer, and we did not examine IgG isotypes. Moreover, considering the initial number of enrolled participants, not all received a third booster dose. However, our study has several strengths. The participants were followed for 90 days after the third booster dose, and there are limited published data relevant to the dynamics of antibodies, especially after five vaccine doses. Moreover, we measured IgA responses after each booster dose, while few studies have investigated the magnitude of these antibody types. Finally, as previously mentioned, many study participants were residents of long-term care facilities, displaying severe comorbidities, namely individuals at high risk for severe/fatal disease, and our findings could contribute to decision making on risk mitigation interventions.

## 5. Conclusions

In conclusion, our study demonstrates that booster vaccination against COVID-19 should be recommended, particularly to individuals at high risk for severe/fatal disease. Moreover, anti-SARS-CoV-2 IgA responses may contribute to improving the prognosis of SARS-CoV-2 infection. Further studies need to be conducted to confirm this hypothesis.

## Figures and Tables

**Table 1:** (A) Magnitude of anti-SARS-CoV-2 IgG responses on day 90 after the first booster dose vaccination. (B) Magnitude of anti-SARS-CoV-2 IgG responses on day 90 after second booster dose vaccination.

(A)							
1st Booster	History of SARS-CoV-2 * Infection						
	Yes (Twice) (N = 7)	Yes (Once) (N = 69)			No (N = 175)		
Parameter	N, Median IgG Levels (AU/mL) (IQR)	N, Median IgG Levels (AU/mL) (IQR **)	*p*-Value	Coefficient, 95% CI ***	N, Median IgG Levels (AU/mL) (IQR)	*p*-Value	Coefficient, 95% CI
Sex (M/F)	M: 4, 72,844 (39,401) F: 3, 40,000 (-)	M: 32, 20,837 (27,342) F: 37, 20,307 (47,811)	0.635	3058 (−9745–15,861)	M: 77, 5420 (9552) F: 98, 6392 (9027)	0.666	−774 (−4302–2754)
Age (years)	Rho: −0.22	Rho: 0.21	0.290	266 (−232–765)	Rho = 0.003	0.817	12.2 (−92.1–116.6)
Diabetes mellitus	No: 3, 80,000 (-) Yes: 4, 52,569 (42,383)	No: 56, 18,656 (30,786) Yes: 13, 23,941 (27,539)	0.608	−4520 (−22,045–13,006)	No: 141, 5939 (9032) Yes: 34, 6780 (9979)	0.800	577 (−3904–5059)
Chronic liver disease	No: 7, 65,688 (40,550)	No: 69, 20,307 (28,326)		-	No: 171, 6102 (8958) Yes: 4, 2640 (6918)	0.378	−5159 (−16,694–6375)
Insomnia/psychiatric diseases	No: 2, 80,000 Yes: 5, 40,000 (37,001)	No: 28, 20,222 (35,584) Yes: 41, 21,457 (26,440)	0.070	−14,984 (−31,243–1275)	No: 103, 5762 (8485) Yes: 72, 6970 (11,959)	0.862	349 (−3607–4304)
Vaccine	Adenovirus vector: 1 Monovalent mRNA: 6, 52,844 (42,354)	Adenovirus vector: 10, 9022 (11,563) Monovalent mRNA: 59, 23,941 (36,924)	0.015	−26,819 (−48,154–−5484)	Adenovirus vector: 31, 3637 (5396) Monovalent mRNA: 144, 6456 (9873)	0.130	−3780 (−8684–1124)
(B)							
2nd Booster	History of SARS-CoV-2 * Infection						
	Yes (Twice) (N = 16)	Yes (Once) (N = 68)			No (N = 42)		
Parameter	N, Median IgG Levels (AU/mL) (IQR **)	N, Median IgG Levels (AU/mL) (IQR)	*p*-Value	Coefficient, 95% CI ***	N, Median IgG Levels (AU/mL) (IQR)	*p*-Value	Coefficient, 95% CI
Sex (M/F)	M: 8, 18,060 (14,802) F: 8, 32,416 (57,477)	M: 29, 40,830 (55,027) F: 39, 40,000 (53,366)	0.486	4979 (−9237–19,196)	M: 22, 7961 (11,382) F: 20, 7256 (21,153)	0.183	5281 (−2615–13,178)
Age (years)	Rho: 0.58	Rho: −0.10	0.721	−89 (−587–408)	Rho: −0.24	0.067	−282 (−584–21)
Chronic heart disease	No: 11, 24,832 (58,061) Yes: 5, 19,793 (42,217)	No: 53, 40,000 (46,164) Yes: 15, 26,492 (68,610)	0.856	−1470 (−17,588–14,648)	No: 28, 7219 (13,533) Yes: 14, 9697 (11,502)	0.415	3189 (−4658–11,035)
Chronic respiratory disease	No: 15, 19,794 (26,763) Yes: 1	No: 61, 40,000 (54613) Yes: 7, 55,676 (53,147)	0.192	15,380 (−7960–38,719)	No: 41, 7992 (11,821) Yes: 1	0.323	−12,040 (−36,454–12,375)
Chronic liver disease	No: 16, 21,124 (49,992) Yes: 0	No: 67, 40,000 (53,147) Yes: 1	0.265	−31,262 (−86,852–24,329)	No: 39, 7919 (11,981) Yes: 3, 8004 (-)	0.285	−7842 (−22,519–6835)
Cancer	Νο: 16, 21,124 (49992) Yes: 0	Νο: 65, 40,000 (52,965) Yes: 3, 26,634 (-)	0.073	−32,848 (−68,816–3119)	Νο: 40, 7998 (11,896) Yes: 2, 2921 (-)	0.114	−14,251 (−32,125–3623)
Vaccine	Bivalent mRNA: 3, 22,454 Monovalent mRNA: 13, 19,794 (42,803)	Bivalent mRNA: 24, 49,241 (51,723) Monovalent mRNA: 44, 40,000 (61,535)	0.488	4873 (−9106–18,851)	Bivalent mRNA: 7, 40,000 (43,746) Monovalent mRNA: 35, 6519 (9949)	0.001	19,244 (8247–30,242)

* SARS-CoV-2, severe acute respiratory syndrome coronavirus 2; ** IQR, interquartile range; *** CI, confidence interval.

**Table 2:** (A) Magnitude of anti-SARS-CoV-2 IgA responses on day 90 after the first booster dose vaccination. (B) Magnitude of anti-SARS-CoV-2 IgA responses on day 90 after the second booster dose vaccination.

(A)							
1st Booster	History of SARS-CoV-2 * Infection						
	Yes (Twice) (N = 7)	Yes (Once) (N = 69)			No (N = 175)		
Parameter	N, Median IgA Levels (U/mL) (IQR **)	N, Median IgA Levels (U/mL) (IQR)	*p*-Value	Coefficient, 95% CI ***	N, Median IgA Levels (U/mL) (IQR)	*p*-Value	Coefficient, 95% CI
Sex (M/F)	M: 4, 35.7 (33.6) F: 3, 39.8 (-)	M: 32, 24.6 (7.4) F: 37, 23.6 (11.9)	0.466	−1.90 (−7.08–3.28)	M: 77, 13.1 (13.4) F: 98, 17.1 (22.1)	0.710	0.71 (−3.05–4.47)
Age (years)	Rho: N/A ****	Rho: 0.11	0.453	−0.07 (−0.25–0.11)	Rho = 0.05	0.514	0.04 (−0.08–0.16)
Thyroid disease	No: 7, 39.8 (28.0)	No: 63,24.2 (7.7) Yes: 6, 18.0 (14.0)	0.079	−8.33 (−17.65–1.00)	No: 154, 15.0 (19.6) Yes: 21, 8.2 (23.8)	0.555	−1.68 (−7.28–3.92)
Chronic liver disease	No: 7, 39.8 (28.0)	No: 69, 23.7 (9.4)	-		No: 171, 14.7 (19.8) Yes: 4, 8.8 (12.0)	0.282	−6.57 (−18.58–5.45)
Other	No: 4, 49.7 (15.0) Yes: 3, 21.8 (-)	No: 39, 24.8 (7.8) Yes: 30, 23.5 (6.6)	0.868	0.49 (−5.41–6.39)	No: 103, 15.9 (21.9) Yes: 72, 13.0 (15.4)	0.063	−4.26 (−8.74–0.23)
Vaccine	Adenovirus vector: 1 Monovalent mRNA: 6, 35.2 (30.1)	Adenovirus vector: 10, 37.3 (39.1) Monovalent mRNA: 59, 23.7 (7.5)	0.401	3.46 (−4.73–11.66)	Adenovirus vector: 31, 5.6 (10.2) Monovalent mRNA: 144, 17.1 (19.3)	<0.001	−9.81 (−14.89–−4.72)
(B)							
2nd Booster	History of SARS-CoV-2 * Infection						
	Yes (Twice) (N = 16)	Yes (Once) (N = 68)			No (N = 41)		
Parameter	N, Median IgA Levels (U/mL) (IQR **)	N, Median IgA Levels (U/mL) (IQR)	*p*-Value	Coefficient, 95% CI ***	N, Median IgA Levels (U/mL) (IQR)	*p*-Value	Coefficient, 95% CI
Sex (M/F)	M: 8, 36.6 (12.6) F: 8, 47.6 (38.9)	M: 29, 41.0 (42.6) F: 39, 38.8 (42.1)	0.229	−6.24 (−16.51–4.03)	M: 22, 16.5 (15.8) F: 19, 16.8 (35.1)	0.583	−3.52 (−16.46–9.43)
Age (years)	Rho: 0.32	Rho: −0.10	0.889	−0.03 (−0.40–0.35)	Rho: −0.02	0.319	−0.34 (−1.02–0.34)
Hypertension	No: 7, 34.7 (16.9) Yes: 9, 45.1 (25.3)	No: 32, 38.9 (35.3) Yes: 36, 39.0 (42.8)	0.051	10.16 (−0.04–20.36)	No: 15, 33.8 (47.1) Yes: 26, 15.2 (17.3)	0.002	−24.10 (−38.62–−9.59)
Dyslipidemia	No: 10, 36.6 (16.5) Yes: 6, 45.2 (32.8)	No: 48, 36.7 (42.3) Yes: 20, 43.2 (39.5)	0.208	6.43 (−3.63–16.32)	No: 28, 15.1 (20.3) Yes: 13, 27.4 (23.5)	0.065	13.72 (−0.91–28.35)
stroke/TIA	No: 16, 42.9 (16.8) Yes: 0	No: 56, 44.4 (42.8) Yes: 12, 28.6 (18.5)	0.048	−12.44 (−24.74–−0.13)	No: 33, 16.7 (26.0) Yes: 8, 18.7 (21.3)	0.812	−1.84 (−17.54–13.86)
Chronic liver disease	No: 16, 42.9 (16.8) Yes: 0	No: 67, 38.8 (42.1) Yes: 1	0.177	25.30 (−11.74–62.35)	No: 38, 18.6 (26.0) Yes: 3, 12.5 (-)	0.147	−19.38 (−45.99–7.23)
Chronic kidney disease	No: 15, 40.7 (18.5) Yes: 1	No: 66, 38.9 (42.1) Yes: 2, 47.6 (-)	0.347	12.48 (−13.85–38.81)	No: 37, 19.8 (25.3) Yes: 4, 6.2 (8.1)	0.720	−3.83 (−25.47–17.81)
Insomnia/psychiatric diseases	No: 2, 25.8 (-) Yes: 14, 45.2 (19.8)	No: 24, 31.3 (43.2) Yes: 44, 44.4 (40.9)	0.870	−0.80 (−10.59 −8.98)	No: 16, 17.1 (22.6) Yes: 25, 16.8 (24.4)	0.667	−2.66 (−15.16–9.84)
Other	No: 9, 38.6 (26.1) Yes: 7, 45.1 (18.5)	No: 29, 49.5 (42.4) Yes: 39, 33.9 (38.1)	0.118	−7.56 (−17.10–1.99)	No: 13, 17.4 (32.1) Yes: 28, 16.7 (24.5)	0.081	14.73 (−1.91–31.36)
Vaccine	BA1&BA4/5: 3, 27.1 BNT162b2: 13, 45.4 (22.9)	BA1&BA4/5: 24, 27.2 (6.32) BNT162b2: 44, 53.2 (36.6)	<0.001	−20.48 (−30.17– −10.79)	BA1&BA4/5: 6, 27.8 (40.8) BNT162b2: 35, 15.6 (22.5)	0.347	8.89 (−10.10–27.87)

* SARS-CoV-2, severe acute respiratory syndrome coronavirus 2; ** IQR, interquartile range; *** CI, confidence interval; Ν/A ****, non applicable.

**Table 3:** Cox regression analysis of participants who received first or second/third booster dose to investigate the impact of the number of booster doses on participants’ survival.

N = 150	*p*-Value	HR *	95.0% CI ** for HR	
			Lower	Upper
Age	0.088	1.03	0.99	1.08
Comorbidities > 2	0.172	2.63	0.66	10.57
Number of booster doses until infection (2/3 vs. 1)	0.712	0.77	0.19	3.07
History of SARS-CoV-2 infection	0.449	0.55	0.11	2.61

* HR, Hazard Ratio, ** CI, confidence interval.

**Table 4:** Binary logistic regression to investigate the effect of age, presence of more than two comorbidities, and number of booster doses on the risk of death due to COVID-19.

N = 177	*p*-Value	OR *	95% CI ** for OR	
			Lower	Upper
Age	0.077	1.05	0.99	1.12
Comorbidities > 2	0.318	2.77	0.38	20.52
Number of booster doses until death (2/3 vs. 1)	0.031	0.08	0.01	0.79

* OR, Odds Ratio, ** CI, confidence interval.

## Data Availability

Data available on request from the corresponding author.

## Supplementary Materials

The following supporting information can be downloaded at: https://www.mdpi.com/article/10.3390/vaccines12010012/s1, Table S1: Overview of demographic and clinical data of COVID-19 vaccinated participants; Table S2: Clinical and laboratory characteristics of vaccinated participants who died from COVID-19; Table S3: Magnitude of IgG anti-SARS-CoV-2 responses on day 90 after the first booster vaccination in relation to comorbidities and history of SARS-CoV-2 infection; Table S4: Magnitude of IgG anti-SARS-CoV-2 responses on day 90 after the second booster vaccination in relation to comorbidities and history of SARS-CoV-2 infection; Table S5: Magnitude of IgG anti-SARS-CoV-2 responses on day 90 after the third booster vaccination (bivalent BA.4/5) in relation to comorbidities and history of SARS-CoV-2 infection; Table S6: Magnitude of IgA anti-SARS-CoV-2 responses on day 90 after the first booster vaccination in relation to comorbidities and history of SARS-CoV-2 infection; Table S7: Magnitude of IgA anti-SARS-CoV-2 responses on day 90 after the second booster vaccination in relation to comorbidities and history of SARS-CoV-2 infection; Table S8: Magnitude of IgA anti-SARS-CoV-2 responses on day 90 after the third booster vaccination (bivalent BA.4/5) in relation to comorbidities and history of SARS-CoV-2 infection; Table S9: Multiple regression analysis of anti-SARS-CoV-2 IgG and IgA responses on day 90 after booster vaccination.
