# Supplementary material for: Dynamics of Anti-SARS-CoV-2 IgA and IgG Responses and Their Protective Effect against Fatal Disease after Booster COVID-19 Vaccination
Source: Vaccines (Basel). 2023 Dec 21;12(1):12. doi: 10.3390/vaccines12010012 (PMC10819547; doi:10.3390/vaccines12010012)
Supplement: Supplementary file 1 [file vaccines-12-00012-s001.zip › Supplementary Table S1_29-11-23.pdf]

Supplementary Table S1. Overview of demographic and clinical data of COVID-19 vaccinated participants.

	Total	mRNA vaccine*	Adenovirus vector-based vaccines*
No.	260	218	42
Gender (male/female)	114/146	89/129	25/17
Age (years; median, range)	68 (26 – 107)	72 (27 – 107)	50 (26 – 84)
Hypertension No. (%)	12 (46.5%)	107 (49.1%)	14 (33.3%)
Diabetes mellitus No. (%)	51 (19.6%)	48 (22.0%)	3 (7.1%)
Dyslipidemia No. (%)	81 (31.2%)	70 (32.1%)	11 (26.2%)
Chronic heart disease ^ No. (%)	55 (21.2%)	53 (24.3%)	2 (4.8%)
Chronic respiratory disease ** No. (%)	11 (4.2%)	10 (4.6%)	1 (2.4%)
Stroke and transient ischemic attacks No. (%)	24 (9.2%)	1 (1.5%)	0 (0.0%)
Thrombotic attacks No. (%)	12 (4.6%)	11 (5.0%)	1 (2.4%)
Chronic liver disease # No. (%)	4 (1.5%)	3 (1.4%)	1 (2.4%)
Chronic kidney disease ^^ No. (%)	11 (0.7%)	11 (5.0%)	0 (0.0%)
Thyroid disease *** No. (%)	29 (11.1%)	22 (10.1%)	7 (16.7%)
Autoimmune/autoinflammatory diseases § No. (%)	16 (6.2%)	16 (7.3%)	0 (0.0%)
Cancer ## No. (%)	9 (3.5%)	9 (4.1%)	0 (0.0%)
Insomnia or psychiatric diseases**** No. (%)	123 (47.3%)	122 (55.9%)	1 (2.4%)
Others ^^^ No. (%)	110 (42.3%)	108 (49.5%)	2 (4.8%)
Comorbidities (at least one) (n, %) No. (%)	212 (81.5%)	188 (86.2%)	24 (57.1%)
Comorbidities (at least two) No. (%)	110 (42.3%)	107 (49.1%)	3 (7.1%)

* mRNA vaccine: BNT162b2 (Comirnaty; Pfizer/BioNtech), adenovirus vector-based vaccines: ChAdOx1 nCoV-10 (Oxford/AstraZeneca, 24 participants) and Ad.26.COV2.S (Johnson & Johnson's Janssen, 18 participants). The type of vaccination refers to the initial two doses (except for those receiving Ad.26.COV2 who vaccinated once); however, all participants received booster doses with BNT162b2 and bivalent BA.1 (Comirnaty Original/Omicron BA.1) or BA.4/5 vaccines (Comirnaty Original/Omicron BA.4-5). ^ Chronic heart disease includes atrial fibrillation, arrhythmias, myocardial infraction, valvular heart disease and coronary heart disease; *Chronic respiratory disease includes chronic obstructive pulmonary disease and asthma; #Chronic liver disease includes primary biliary cirrhosis and alcohol-related liver disease; ^^Chronic kidney disease includes chronic renal insufficiency; **Thyroid disease includes hypothyroidism (non-autoimmune), multinodular goiter and Hashimoto disease; §Autoimmune/autoinflammatory disease includes rheumatoid arthritis, ancylosing spondylarthritis, scleroderma, psoriasis, lupus; ## Cancer includes patients with history of breast, uterus and prostate cancer and hematologic malignancies (leukemias, myelodysplasia, polycythemia vera); *** Insomnia or psychiatric diseases includes patients with insomnia, schizophrenia, depression, bipolar disorders and psychosis ^^^ Others includes prostate hyperplasia, vision and hearing disorders, chronic ethylation, Down syndrome, osteoporosis, spastic quadriplegia, history of epilepsy, anemia, pyramidal tract disorders, hyperuricemia, Parkinson’s disease, stomach ulcer, esophagitis, diverticulitis, hydrocephalus, gout, adrenal hyperplasia, Pick disease, Buerger disease, osteoarthritis.
