# Supplementary material for: Dynamics of Anti-SARS-CoV-2 IgA and IgG Responses and Their Protective Effect against Fatal Disease after Booster COVID-19 Vaccination
Source: Vaccines (Basel). 2023 Dec 21;12(1):12. doi: 10.3390/vaccines12010012 (PMC10819547; doi:10.3390/vaccines12010012)
Supplement: Supplementary file 1 [file vaccines-12-00012-s001.zip › Supplementary Table S2_29-11-23.pdf]

Supplementary Table S2. Clinical and laboratory characteristics of vaccinated participants who died from COVID-19

No	Age	Sex	Medical history – comorbidities	Antibody responses on day 90 after vaccination *			Comments
				1st and 2nd dose (BNT162b2)	1st booster dose (BNT162b2)	2nd booster dose (BNT162b2)	
#477	62	Female	Systemic scleroderma, atrial fibrillation, hypothyroidism, osteoporosis, epilepsy	IgG 134.6 AU/mL, IgA 1.65 U/mL	Non-estimated		SARS-CoV-2 infection and death before day 90 after the 1st booster vaccination
#359	100	Female	Hypertension, diabetes mellitus, hypothyroidism, depression	IgG 1,534.1 AU/mL, IgA 7.63 U/mL	IgG 10,083.1 AU/mL, IgA 74.81 U/mL		SARS-CoV-2 infection and death 173 days after booster vaccination
#389	70	Male	Parkinson's disease, mental retardation, organic psychosis, prostate hyperplasia	IgG 202.0 AU/mL, IgA 36.41 U/mL	IgG 1,907.3 AU/mL, IgA 14.71 U/mL		SARS-CoV-2 infection and death 260 days after booster vaccination
#354	65	Female	Down's syndrome, dyslipidemia, osteoporosis	IgG 737.4 AU/mL, IgA 9.93 U/mL	IgG 3,203.9 AU/mL, IgA 43.61 U/mL		1st SARS-CoV-2 infection 127 days after booster vaccination; 2nd SARS-CoV-2 infection and death 389 days after booster vaccination
#459	93	Female	Dementia, osteoarthritis	IgG 553.0 AU/mL, IgA 3.12 U/mL	IgG 7,312.4 AU/mL, IgA 21.23 U/mL	IgG 11,461.0 AU/mL, IgA 41.07 U/mL	Disease and death 112 days after the 2nd booster vaccination

* The cut-off values for antibody responses after vaccination were 50 AU/mL for IgG anti-SARS-CoV-2 levels and 10 U/mL for IgA anti-SARS-CoV-2 levels.
