# Supplementary material for: Dynamics of Anti-SARS-CoV-2 IgA and IgG Responses and Their Protective Effect against Fatal Disease after Booster COVID-19 Vaccination
Source: Vaccines (Basel). 2023 Dec 21;12(1):12. doi: 10.3390/vaccines12010012 (PMC10819547; doi:10.3390/vaccines12010012)
Supplement: Supplementary file 1 [file vaccines-12-00012-s001.zip › Supplementary Table S3_29-11-23.pdf]

Supplementary Table S3. Magnitude of IgG anti-SARS-CoV-2 responses on day 90 after the first booster vaccination in relation to comorbidities and history of SARS-CoV-2 infection.

**Parameter**	**No, IgG levels (AU/mL) (Median, IQR)**	**p-value (Univariate analysis)**	**p-value (Multivariate analysis)**	**Coefficient (95% CI)**
Sex (male/female)	Male: 113, 7,873, 18,099<br>Female: 138, 8,656, 14,570	0.726	0.926	-207.4 (-4,602.0 – 4,187.3)
Age (years)	Rho = 0.09	0.141	0.218	86.7 (-51.7 – 225.1)
Comorbidities (no VS ≥ 1)	No: 49, 8,007, 11,802<br>≥ 1: 202, 8,451, 18,476	0.998		
Comorbidities (1 VS ≥ 2)	one: 148, 6,839, 13,674<br>≥ 2: 103, 10,573, 21,481	0.150		
Hypertension	No: 135, 6,500, 16,711<br>Yes:116, 10,428, 17,808	0.303		
Diabetes Mellitus	No: 200, 7,654, 14,272<br>Yes: 51, 11,600, 20,640	0.105	0.959	-145.5<br>(-5,753.9 – 5,462.9)
Dyslipidemia	No: 173, 8,320, 16,086<br>Yes: 78, 8,597, 17,952	0.814		
Chronic heart disease	No: 200, 8,251, 14,356<br>Yes: 51, 10,572, 19,232	0.314		
Stroke/TIA	No: 228, 8,371, 17,234<br>Yes: 23, 8,742, 9,372	0.392		
Venous/arterial thromboses	No: 241, 8,182, 14,741<br>Yes: 10, 17,425, 20,071	0.266		
Chronic respiratory disease	No: 240, 8,370, 15,116<br>Yes: 11, 10,369, 30,700	0.936		
Autoimmune/ autoinflammatory disorders	No: 236, 8,436, 15,073<br>Yes: 15, 7,584, 19,750	0.687		
Thyroid disease	No: 224, 8,495, 15,073<br>Yes: 27, 7,056, 26,589	0.518		

Chronic liver disease	No: 247, 8,451, 16,589<br>Yes: 4, 2,639, 6,918	0.084	0.534	-5,471.2<br>(-22,770.8 – 1,390.1)
Chronic kidney disease	No: 240, 8,370, 14,650<br>Yes: 11, 10,935, 30,194	0.880		
Cancer	No: 242, 8,251, 14,688<br>Yes: 9, 23,879, 28,033	0.387		
Insomnia/psychiatric diseases	No: 133, 6,910, 11,354<br>Yes: 118, 10,530, 21,575	0.167	0.153	-3,705.1<br>(-8,800.3 – 1,390.1)
Others	No: 146, 7,939, 14,921<br>Yes: 105, 8,857, 18,117	0.844		
History of SARS-CoV-2 infection	No: 175, 5,942, 8,987<br>Yes (once): 69, 20,307, 28,326<br>Yes (twice): 7, 65,688, 40,550	**<0.001**	**<0.001**	21309.6<br>(17151.2 – 25468.0)
Vaccination type (adenovirus vector-based VS mRNA vaccines)	Adenovirus vector-based:<br>42, 4,453, 7,349<br>mRNA: 209, 9,903, 18,693	**0.002**	**0.011**	-8,322.7<br>(-14,760.5 – -1,884.9)

Abbreviations: IQR, interquartile range; TIA, transient ischemic attack; 95%CI, 95% confidence interval
