# Supplementary material for: Dynamics of Anti-SARS-CoV-2 IgA and IgG Responses and Their Protective Effect against Fatal Disease after Booster COVID-19 Vaccination
Source: Vaccines (Basel). 2023 Dec 21;12(1):12. doi: 10.3390/vaccines12010012 (PMC10819547; doi:10.3390/vaccines12010012)
Supplement: Supplementary file 1 [file vaccines-12-00012-s001.zip › Supplementary Table S4_29-11-23.pdf]

Supplementary Table S4. Magnitude of IgG anti-SARS-CoV-2 responses on day 90 after the second booster vaccination in relation to comorbidities and history of SARS-CoV-2 infection

Parameter	No, IgG levels (AU/mL) (Median, IQR)	p-value (Univariate analysis)	p-value (Multivariate analysis)	Coefficient (95% CI)
Sex (male/female)	Male: 60, 18,290, 34,026<br>Female: 67, 30,590, 57,280	0.206	0.405	3,060 (-5,194 – 12,781)
Age (years)	Rho = -0.04	0.683	0.346	154 (-168 – 477)
Comorbidities (No VS ≥ 1)	No: 6, 27,617, 49,499<br>≥ 1: 121, 26,732, 45,666	0.690		
Comorbidities (1 VS ≥ 2)	one: 60, 27,617, 35,573<br>≥ 2: 67, 26,634, 62,229	0.864		
Hypertension	No: 55, 27,507, 42,439<br>Yes: 72, 26,683, 55,293	0.396		
Diabetes mellitus	No: 95, 26,853, 45,607<br>Yes: 32, 28,300, 40,259	0.942		
Dyslipidemia	No: 87, 27,728, 38,226<br>Yes: 40, 24,557, 60,279	0.890		
Chronic heart disease	No: 92, 34,850, 45,494<br>Yes: 35, 16,020, 39,097	0.181	0.441	-3,951 (-14,080 – 6,177)
Stroke/TIA	No: 107, 26,853, 46,358<br>Yes: 20, 26,287, 42,287	0.461		
Venous/arterial thromboses	No: 119, 27,016, 54,330<br>Yes: 8, 20,424, 32,985	0.392		
Chronic respiratory disease	No: 118, 25,662, 38,573<br>Yes: 9, 55,676, 53,257	0.092	0.285	9,558 (-8,050 – 27,167)
Autoimmune/ autoinflammatory disorders	No: 115, 27,216, 54,057<br>Yes: 12, 15,086, 34,556	0.252		
Thyroid disease	No: 118, 27,035, 45,445<br>Yes: 9, 26,634, 51,635	0.770		

Chronic liver disease	No: 123, 27,507, 46,631<br>Yes: 4, 11,396, 15,392	0.089	0.272	-14,301 (-39,989 – 11,386)
Chronic kidney disease	No: 119, 26,853, 44,559<br>Yes: 8, 25,164, 58,136	0.462		
Cancer	No: 122, 27,361, 48,395<br>Yes: 5, 8,871, 28,166	0.082	0.400	-14,301 (-39,989 – 11,386)
Insomnia/psychiatric diseases	No: 133, 6,910, 11,354<br>Yes: 118, 10,530, 21,575	0.812		
Others	No: 146, 7,939, 14,921<br>Yes: 105, 8,857, 18,117	0.658		
History of SARS-CoV-2 infection	No: 42, 7,955, 11,992<br>Yes (once): 68, 40,000, 53,311<br>Yes (twice): 16, 21,124, 49,992	**<0.001**	**<0.001**	14,512 (7,944 – 21,081)
Vaccination type (bivalent BA.1 & BA.4/5 VS monovalent mRNA BNT162b2 vaccines)	BA1 & BA4/5: 34, 40,000, 45,233<br>BNT162b2: 93, 17,917, 33,354	**0.002**	**0.006**	-14,712 (4,405 – 25,019)

Abbreviations: IQR, interquartile range; TIA, transient ischemic attack; 95%CI, 95% confidence interval
