# Supplementary material for: Dynamics of Anti-SARS-CoV-2 IgA and IgG Responses and Their Protective Effect against Fatal Disease after Booster COVID-19 Vaccination
Source: Vaccines (Basel). 2023 Dec 21;12(1):12. doi: 10.3390/vaccines12010012 (PMC10819547; doi:10.3390/vaccines12010012)
Supplement: Supplementary file 1 [file vaccines-12-00012-s001.zip › Supplementary Table S5_29-11-23.pdf]

Supplementary Table S5. Magnitude of IgG anti-SARS-CoV-2 responses on day 90 after the third booster vaccination (bivalent BA.4/5) in relation to comorbidities and history of SARS-CoV-2 infection.

Parameter	N, IgG levels (AU/mL) (Median, IQR)	p-value (Univariate analysis)	p-value (Multivariate analysis)	Coefficient (95% CI)
Sex (male)	Male: 15, 20,776, 29,267<br>Female: 19, 26,636, 47,449	0.242	0.517	6,342 (-13,434 – 26,120)
Age (years)	Rho=-0.05	0.781	0.643	-199 (-1,072 – 673)
Comorbidity (no VS >= 1)	No:0<br>Yes: 34, 25,783, 36,253	-		
Comorbidity (0 VS >=2)	No: 21, 23,228, 33,679<br>Yes: 13, 26,219, 39,993	0.576		
Hypertension	No: 14, 26,907, 29,820<br>Yes: 20, 25,783, 46,314	0.823		
Diabetes Mellitus	No: 27, 23,789, 40,760<br>Yes: 7, 26,219, 8,282	0.934		
Dyslipidemia	No: 17, 26,637, 31,544<br>Yes: 17, 23,758, 35,332	0.394		
Chronic heart disease	No: 27, 23,789, 35,706<br>Yes: 7, 26,219, 31,247	0.588		
stroke/ TIA	No: 32, 25,783, 37,564<br>Yes: 2, 14,842, -	-		
venous/ arterial thromboses	No: 33, 40,000, 5,636<br>Yes: 1	-		
Chronic respiratory disease	No: 33, 25,353, 33,679<br>Yes: 1	-		
Autoimmune/ autoinflammatory disorders	No: 32, 24,571, 38,483<br>Yes: 2, 38,656, -	-		
Thyroid disease	No: 31, 25,353, 40,760<br>Yes: 3, 30,586, -	-		
Chronic liver disease	No: 34, 25,783, 36,253<br>Yes:0	-		
Chronic kidney disease	No: 33, 25,353, 33,679<br>Yes: 1	-		
Cancer	No: 34, 25,783, 36,253<br>Yes: 0	-		
Chronic kidney disease and cancer	No:33, 25,353, 33,679<br>Yes:1	-		
Insomnia/ psychiatric diseases	No: 10, 26,445, 52,903<br>Yes: 24, 25,783, 33,715	0.724		
Other	No: 16, 26,216, 60,924<br>Yes: 18, 24,571, 29,169	0.695		
History of SARS-CoV-2 infection	No: 14, 17,710, 29,617<br>Yes (once): 18, 28,611, 39,283<br>Yes (twice): 2, 39,118, -	0.094 (No VS once)	0.308	8,549 (-8,279 – 25,377)

Abbreviations: IQR, interquartile range; 95%CI, 95% confidence interval
