# Supplementary material for: Dynamics of Anti-SARS-CoV-2 IgA and IgG Responses and Their Protective Effect against Fatal Disease after Booster COVID-19 Vaccination
Source: Vaccines (Basel). 2023 Dec 21;12(1):12. doi: 10.3390/vaccines12010012 (PMC10819547; doi:10.3390/vaccines12010012)
Supplement: Supplementary file 1 [file vaccines-12-00012-s001.zip › Supplementary Table S6_29-11-23.pdf]

Supplementary Table S6. Magnitude of IgA anti-SARS-CoV-2 responses on day 90 after the first booster vaccination in relation to comorbidities and history of SARS-CoV-2 infection

**Parameter**	**No, IgG levels (U/mL) (Median, IQR)**	**p-value (Univariate analysis)**	**p-value (Multivariate analysis)**	**Coefficient (95% CI)**
Sex (male/female)	Male: 113, 13.8, 17.2<br>Female: 138, 21.0, 21.1	0.777	0.836	-32.0 (-2.74 – 3.38)
Age (years)	Rho = 0.07	0.285	0.971	-0.02 (-10.0 – 0,09)
Comorbidities (no VS ≥ 1)	No: 49, 26.8, 19.8<br>≥ 1: 202, 18.7, 16.8	**0.018**		
Comorbidities (1 VS ≥ 2)	one: 148, 18.7, 19.9<br>≥ 2: 103, 19.8, 17.5	0.291		
Hypertension	No: 135, 20.8, 20.2<br>Yes:116, 18.3, 18.2	0.364		
Diabetes mellitus	No: 200, 20.0, 19.6<br>Yes: 51, 18.8, 16.9	0.678		
Dyslipidemia	No: 173, 18.8, 19.2<br>Yes: 78, 20.7, 18.6	0.528		
Chronic heart disease	No: 200, 18.9, 19.2<br>Yes: 51, 20.8, 19.4	0.315		
Stroke/TIA	No: 228, 19.9, 19.8<br>Yes: 23, 17.9, 12.4	0.364		
Venous/arterial thromboses	No: 241, 19.2, 19.5<br>Yes: 10, 21.8, 13.4	0.684		
Chronic respiratory disease	No: 240, 19.7, 19.0<br>Yes: 11, 11.2, 22.3	0.306		
Autoimmune/ autoinflammatory disorders	No: 236, 19.2, 19.4<br>Yes: 15, 20.8, 13.5	0.901		
Thyroid disease	No: 224, 20.6, 17.9<br>Yes: 27, 12.2, 21.5	**0.019**	0.166	-3.42 (-8.27 – 1.43)

Chronic liver disease	No: 247, 19.8, 19.1<br>Yes: 4, 8.8, 12.0	0.057	0.227	-7.24 (-19.01 – 4.53)
Chronic kidney disease	No: 240, 19.7, 19.0<br>Yes: 11, 14.3, 21.1	0.202		
Cancer	No: 242, 19.5, 19.4<br>Yes: 9, 18.2, 20.2	0.901		
Insomnia/psychiatric diseases	No: 133, 17.9, 20.4<br>Yes: 118, 20.9, 16.0	0.509		
Others	No: 146, 22.1, 20.9<br>Yes: 105, 17.9, 14.1	0.113	0.063	-3.37 (-6.91 – 0.18)
History of SARS-CoV-2 infection	No: 175, 14.2, 19.8<br>Yes (once): 69, 23.7, 9.4<br>Yes (twice): 7, 38.8, 28.0	**<0.001**	**<0.001**	8.79 (6.00 – 11.58)
Vaccination type (adenovirus vector-based VS mRNA vaccines)	Adenovirus vector-based: 42, 21.4, 17.4<br>mRNA: 209, 8.5, 22.1	**<0.001**	**0.006**	-6.07<br>(-10.36 – -1.78)

Abbreviations: IQR, interquartile range; TIA, transient ischemic attack; 95%CI, 95% confidence interval
