# Supplementary material for: Dynamics of Anti-SARS-CoV-2 IgA and IgG Responses and Their Protective Effect against Fatal Disease after Booster COVID-19 Vaccination
Source: Vaccines (Basel). 2023 Dec 21;12(1):12. doi: 10.3390/vaccines12010012 (PMC10819547; doi:10.3390/vaccines12010012)
Supplement: Supplementary file 1 [file vaccines-12-00012-s001.zip › Supplementary Table S7_29-11-23.pdf]

Supplementary Table S7. Magnitude of IgA anti-SARS-CoV-2 responses on day 90 after the second booster vaccination in relation to comorbidities and history of SARS-CoV-2 infection.

**Parameter**	**No, IgA levels (U/mL) (Median, IQR)**	**p-value (Univariate analysis)**	**p-value (Multivariate analysis)**	**Coefficient (95% CI)**
Sex (male/female)	Male: 60, 31.2, 27.2<br>Female: 67, 36.0, 40.8	0.362	0.256	4.13 (-3.04 – 11.29)
Age (years)	Rho = -0.09	0.347	0.762	0.05 (-0.26 – 0.35)
Comorbidities (no VS ≥ 1)	No: 6, 22.3, 44.7<br>≥ 1: 121, 33.9, 32.5	0.378		
Comorbidities (1 VS ≥ 2)	one: 60, 34.7, 40.9<br>≥ 2: 67, 30.7, 26.4	0.527		
Hypertension	No: 55, 38.2, 29.8<br>Yes: 72, 28.4, 37.2	0.152	0.336	-3.80 (-11.58 – 3.99)
Diabetes mellitus	No: 95, 34.6, 32.7<br>Yes: 32, 28.6, 36.8	0.545		
Dyslipidemia	No: 87, 30.6, 34.7<br>Yes: 40,38.2, 33.1	0.132	0.259	4.41 (-3.29 – 12.11)
Chronic heart disease	No: 92, 34.1, 35.0<br>Yes: 35, 31.8, 33.2	0.892		
Stroke/TIA	No: 107, 34.3, 37.9<br>Yes: 20, 27.1, 20.3	0.138	0.435	-3.89 (-13.74 – 5.96)
Venous/arterial thromboses	No: 119, 32.9, 32.1<br>Yes: 8, 36.3, 37.2	0.928		
Chronic respiratory disease	No: 118, 33.9, 33.4<br>Yes: 9, 30.5, 28.8	0.880		
Autoimmune/ autoinflammatory disorders	No: 115, 34.0, 35.9<br>Yes: 12, 27.2, 27.0	0.337		
Thyroid disease	No: 118, 34.1, 32.5<br>Yes: 9, 26.9, 40.1	0.287		

Chronic liver disease	No: 123, 34.0, 35.2<br>Yes: 4, 13.5, 37.5	0.082	0.725	-3.67 (-24.35 – 17.00)
Chronic kidney disease	No: 119, 34.1, 34.8<br>Yes: 8, 18.7, 36.4	0.077	0.076	-13.78 (-29.03 – 1.48)
Cancer	No: 122, 34.0, 33.0<br>Yes: 5, 26.1, 48.0	0.218		
Insomnia/psychiatric diseases	No: 133, 27.4, 37.0<br>Yes: 118, 34.7, 35.2	0.153	0.573	-2.33 (-10.50 – 5.84)
Others	No: 146, 38.8, 43.1<br>Yes: 105, 28.6, 24.4	0.166	0.551	-2.47 (-10.68 – 5.73)
History of SARS-CoV-2 infection	No: 42, 16.8, 24.0<br>Yes (once): 68, 38.9, 42.1<br>Yes (twice): 16, 42.9 16.8	**<0.001**	**<0.001**	11.05 (5.58 – 16.52)
Vaccination type (bivalent BA.1 & BA. 4/5 VS monovalent mRNA vaccines)	BA1 & BA4/5: 34, 27.3, 6.2<br>BNT162b2: 93, 38.7, 47.3	0.062	**0.004**	-12.91 (-21.58 – -4.23)

Abbreviations: IQR, interquartile range; TIA, transient ischemic attack; 95%CI, 95% confidence interval
