# Supplementary material for: Dynamics of Anti-SARS-CoV-2 IgA and IgG Responses and Their Protective Effect against Fatal Disease after Booster COVID-19 Vaccination
Source: Vaccines (Basel). 2023 Dec 21;12(1):12. doi: 10.3390/vaccines12010012 (PMC10819547; doi:10.3390/vaccines12010012)
Supplement: Supplementary file 1 [file vaccines-12-00012-s001.zip › Supplementary Table S8_29-11-23.pdf]

Supplementary Table S8. Magnitude of IgA anti-SARS-CoV-2 responses on day 90 after the third booster vaccination (bivalent BA.4/5) in relation to comorbidities and history of SARS-CoV-2 infection.

**Parameter**	**N, IgA levels (U/mL) (Median, IQR)**	**p-value, (Univariate analysis)**	**p-value (Multivariate analysis)**	**Coefficient (95% CI)**
Sex (male)	Male: 15, 41.8, 57.0<br>Female: 19, 65.8, 29.7	0.242	0.264	9.82 (-7.81 – 27.45)
Age (years)	Rho=-0.15	0.398	0.467	-0.28 (-1.06 – 0.50)
Comorbidity (no VS >= 1)	No:0<br>Yes: 34, 61.2, 37.7	-		
Comorbidity (0 VS >=2)	No: 21, 64.5, 32.2<br>Yes: 13, 41.8, 43.7	0.576		
Hypertension	No: 14, 66.9, 31.8<br>Yes: 20, 58.0, 52.1	0.823		
Diabetes mellitus	No: 27, 64.5, 33.4<br>Yes: 7, 38.7, 43.2	0.934		
Dyslipidemia	No: 17, 63.9, 42.2<br>Yes: 17, 57.4, 40.3	0.394		
Chronic heart disease	No: 27, 64.5, 31.8<br>Yes: 7, 27.9, 54.9	0.588		
Stroke/ TIA	No: 32, 64.2, 42.5<br>Yes: 2, 39.5, -	-		
Venous/ arterial thromboses	No: 33, 63.9, 40.3<br>Yes: 1	-		
Chronic respiratory disease	No: 33, 58.5, 39.3<br>Yes: 1	-		
Autoimmune/ autoinflammatory disorders	No: 32, 64.2, 42.9<br>Yes: 2, 39.3, -	-		
Thyroid disease	No: 31, 63.9, 36.2<br>Yes: 3, 38.7, -	-		
Chronic liver disease	No: 34, 61.2, 37.7<br>Yes:0	-		
Chronic kidney disease	No: 33, 63.9, 34.9<br>Yes: 1	-		
Cancer	No: 34, 61.2, 37.7<br>Yes: 0	-		
Insomnia/ psychiatric diseases	No: 10, 61.0, 36.6<br>Yes: 24, 61.2, 43.5	0.724		
Other	No: 16, 64.2, 36.0<br>Yes: 18, 58.0, 44.1	0.695		
History of SARS-CoV-2 infection	No: 14, 42.2, 56.3<br>Yes (once): 18, 67.4, 29.7<br>Yes (twice): 2, 50.7, -	0.094	0.356	6.89 (-8.11 – 21.89)

Abbreviations: IQR, interquartile range; 95%CI, 95% confidence interval
