# Supplementary material for: Dynamics of Anti-SARS-CoV-2 IgA and IgG Responses and Their Protective Effect against Fatal Disease after Booster COVID-19 Vaccination
Source: Vaccines (Basel). 2023 Dec 21;12(1):12. doi: 10.3390/vaccines12010012 (PMC10819547; doi:10.3390/vaccines12010012)
Supplement: Supplementary file 1 [file vaccines-12-00012-s001.zip › Supplementary Table S9_29-11-23.pdf]

Supplementary Table S9. Multiple regression analysis of anti-SARS-CoV-2 IgG and IgA responses on day 90 after booster vaccination doses..

	**First booster vaccination**		**Second booster vaccination**		**Third booster vaccination**	
	**(No 251)**		**(No 126)**		**(No 34)**	
	***p*-value**	**Coeff. (95% CI)**	***p*-value**	**Coeff. (95% CI)**	***p*-value**	**Coeff. (95% CI)**
**anti-SARS-CoV-2 IgG levels**						
Sex (male/female)	0.866	372 (-3,978 – 4,723)	0.252	5,164 (-3,727 – 14,055)	0.501	6,697 (-13,425 – 26,820)
Age	0.249	80 (-57 – 217)	0.488	117 (-216 – 450)	0.602	-230 (-1,125 – 644)
Comorbidities (≥ 1VS 0-ref)	0.281	-2,739 (-7,734 – 2,256)	0.974	-152 (-9,419 – 9,116)	NA **	
History of SARS-CoV-2 infection	**<0.001**	21,021 (16,939 – 25,103)	**<0.001**	15,257 (8,736 – 21,779)	0.330	8,281 (-8,830 – 25,393)
Vaccine type *	**0.018**	-7,492 (-13,681 – -1,302)	**0.003**	15,580 (5,519 – 25,642)	NA ***	
**anti-SARS-CoV-2 IgA levels**						
Sex (male/female)	0.795	0.40 (-3.40 – 2.61)	0.184	4.98 (-2.39 – 12.36)	0.264	9.82 (-7.81 – 27.45)
Age	0.302	-0.06 (-0.05 – 0.16)	0.332	-0.14 (-0.43 – 0.15)	0.467	-0.28 (-1.06 – 0.50)
Comorbidities (≥ 1VS 0-ref)	**0.003**	-7.26 (-11.95 – -2.57)	0.768	3.20 (-18.21 – 24.60)	NA **	
History of SARS-CoV-2 infection	**<0.001**	8.76 (5.99 – 11.53)	**<0.001**	11.21 (5.89 – 16.52)	0.356	6.89 (-8.11 – 21.89)
Vaccine type *	**0.004**	-6.17 (-10.32 – -2.03)	**0.013**	-10.90 (-19.47 – -2.34)	NA ***	

Abbreviations: CI, confidence intervals; NA, non-available; Coeff., coefficient.
*The comparison of vaccine type concerns the adenovirus vector-based vaccines VS monovalent mRNA of the initial vaccination before the first booster dose, and the bivalent vaccines (BA.1 or BA.4/5) VS monovalent after the second booster dose.
**All vaccinated individuals with the third booster dose displayed at least one comorbidity.
***All individuals vaccinated by the bivalent BA.4/5 vaccine.
